# A nationwide pharmacovigilance investigation on trends and seriousness of adverse events induced by anti-obesity medication

**DOI:** 10.7189/jogh.13.04095

**Published:** 2023-09-01

**Authors:** Yeo Jin Choi, Chang-Young Choi, Choong Ui Kim, Sooyoung Shin

**Affiliations:** 1 Department of Pharmacy, College of Pharmacy, Kyung Hee University, Seoul, South Korea; 2 Department of Regulatory Science, Graduate School, Kyung Hee University, Seoul, South Korea; 3 Institute of Regulatory Innovation through Science (IRIS), Kyung Hee University, Seoul, South Korea; 4 Department of Internal Medicine, Ajou University Medical Hospital, Suwon, South Korea; 5 Department of Pharmacy, College of Pharmacy, Ajou University, Suwon, South Korea; 6 Research Institute of Pharmaceutical Science and Technology (RIPST), Ajou University, Suwon, South Korea

## Abstract

**Introduction:**

Despite rising concerns regarding the safety of anti-obesity medications, there is a lack of comprehensive pharmacovigilance investigations utilising real-world data. We aimed to characterise the prevalence and seriousness of adverse drug events (ADEs) related to anti-obesity medications and to identify predictors associated with increased risk of serious adverse events (SAE), thereby conveying evidence on drug safety.

**Methods:**

We conducted a cross-sectional analysis on ADE cases spontaneously reported to the Korea Adverse Event Reporting System Database (KIDS-KD). ADE reports pertaining to anti-obesity medications prescribed for overweight, obesity (International Classification of Disease, 10th revision (ICD-10) code E66) and abnormal weight gain (ICD-10 code E63.5) were included in the analysis. We performed a disproportionality to detect the association of the system organ class-based ADEs with their seriousness an individual’s sex by estimating reporting odds ratios (RORs) and their 95% confidence intervals (CIs). We performed logistic regression to investigate factors that are substantially associated with increased SAE risks by estimating odds ratio (OR) and their 95% CIs.

**Results:**

The most common causative anti-obesity medication was phentermine, followed by liraglutide. ADEs associated with psychiatric disorders (ROR = 1.734; 95% CI = 1.111-2.707), liver and biliary system disorders (ROR = 22.948; 95% CI = 6.613-70.635), cardiovascular disorders (ROR = 5.707; 95% CI = 1.965-16.574), and respiratory disorders (ROR = 4.567; 95% CI = 1.774-11.762) were more likely to be serious events. Additionally, men are more likely to experience ADEs related gastrointestinal disorders (ROR = 1.411) and less likely to have heart and rhythm disorders (ROR = 0.507). The risk of SAE incidences was positively correlated with being male (OR = 2.196; 95% CI = 1.296-3.721), dual or triple combination of anti-obesity medications (OR = 3.258; 95% CI = 1.633-6.501 and OR = 8.226; 95% CI = 3.046-22.218, respectively), and concomitant administration of fluoxetine (OR = 5.236; 95% CI = 2.218-12.365).

**Conclusions:**

Seriousness of anti-obesity medication-related ADEs differs among system-organ class, while sex-related differences in ADE profiles are also present. The predictors substantially increasing risk of SAE incidences include being male, having a higher number of concomitant medications (including multiple combination of anti-obesity medications), and concurrent use of fluoxetine. Nonetheless, further pharmacovigilance investigation and monitoring are needed to enhance awareness on ADEs induced by anti-obesity medications.

Obesity, characterised as abnormal or excessive fat accumulation, is a significant global health concern, affecting more than 650 million adults [1,2]. It has grown into an epidemic in many countries, such as the USA and South Korea, due to the growing number of obese individuals (including children and adolescents) each year [3]. Consequently, obesity imposes a considerable burden on health care systems and contributes to global morbidity and mortality, alongside chronic complications such as type 2 diabetes mellitus (T2DM), hypertension, dyslipidaemia, coronary artery disease, gastrointestinal (GI) complications, cancer, and chronic respiratory disease [4]. The United States Centers for Disease Control and Prevention (CDC) estimated that there are more than 684 000 obesity-associated cancer cases annually in the USA [5]. Moreover, a recent cohort study found that obese patients are at a greater risk of all-cause mortality than normal-weight patients, and that mortality risk subsequently increases with rising body mass index (BMI) [6]. Obesity was also found to be associated with low quality of life and mental health impairments, as shown by higher uses of psychopharmacological agents for depression and anxiety among obese individuals [7,8]. Hence, implementation of appropriate obesity treatment, inducing substantial weight reductions, are key to improving clinical outcomes of obese populations.

The current American Association of Clinical Endocrinologist and American College of Endocrinology obesity treatment guidelines recommend lifestyle therapy involving behaviour intervention, and changes in diet and physical activity as the primary treatment modality for obesity management [9,10]. Pharmacological treatment, however, is usually reserved for those who failed to lose weight with lifestyle therapy [9]. Although anti-obesity medications induce substantial weight loss compared to lifestyle therapy alone, concerns on their chronic long-term use persist due to safety issues involving cardiovascular and psychiatric effects manifested as suicidal ideation or drug dependence, as suggested by 25 post-marketing withdrawal cases of anti-obesity medications between 1964 and 2009 [11-13]. Currently, there are only five medications approved by the Food and Drug Administration (FDA) for long-term obesity management: liraglutide, phentermine and topiramate, naltrexone and bupropion, semaglutide, and orlistat [9].

Safety concerns regarding anti-obesity medications continues to grow, necessitating clinical trials to discover adverse effects (AEs) induced by these agents. However, there are few pharmacovigilance investigations, referred to as science and activities of detection, assessment, understanding, and prevention of adverse effects, on diverse anti-obesity medications using real-world data (RWD) [12,14]. RWD refers to the data pertaining to patient health status routinely collected from variety sources; for example, one such source used for post-marketing pharmacovigilance assessments is a voluntary adverse drug reaction (ADR) reporting system constructed to ensure patient safety [15-17]. The substantial benefits of RWD utilisation in pharmacovigilance investigation include large-scaled post-marketing surveillance, detection of ADR with rare incidences, and generation of real-world evidence (RWE) on drug safety [18]. We thus aimed to characterise the prevalence and seriousness of adverse drug events (ADEs) related to anti-obesity medications and to identify factors associated with serious ADEs by using a nationwide voluntary ADE reporting system to convey evidence on safety of commonly prescribed anti-obesity agents.

## METHODS

### Data source and definition

We conducted this cross-sectional following the Strengthening the reporting of observational studies in epidemiology (STROBE) guideline [19]. We analysed ADE records spontaneously reported from January 2010 to December 2019 to the Korea Institute of Drug Safety & Risk Management (KIDS) – Korea Adverse Event Reporting System (KAERS) Database (KIDS-KD). KIDS-KD is available to the public and health care professionals (including doctors, nurses, and pharmacists), and any ADE reports submitted to the KIDS-KD are further verified by multiple health care professionals appointed by the Korean Institute of Drug Safety & Risk Management (Ministry of Food and Drug Safety) based on patient’s medical charts, interviews with patient or health care professional, and scientific pharmacovigilance data from manufacturers to minimise biases [20,21]. We evaluated any ADE reports caused by anti-obesity medications commonly prescribed for obesity treatment in South Korea: amfepramone (diethylpropion) [22], betahistine [23], bupropion and naltrexone [9], liraglutide [9], lorcaserin [24], mazindol [25], orlistat [9], phentermine [9], sibutramine [26], and topiramate [9,23,27,28]. Any cases reported as ADEs induced by “other obesity agents” or “centrally acting anti-obesity agents” were excluded from the analysis to ensure the validity of medication-specific ADE analysis. Moreover, we only included ADE cases related to obesity management, as some anti-obesity medications such as liraglutide and topiramate may be prescribed for other diseases. The prespecified diagnostic codes for obesity management include overweight and obesity (E66) and abnormal weight gain (E63.5), per the International Classification of Disease, 10^th^ revision (ICD-10). We extracted the following information from the KIDS-KD: patient demographic information (such age and sex), medical history (including comorbid conditions and diagnostic code for obesity management), information of medication administrations, ADE information with causality, and seriousness of ADE (including hospitalisation, mortality, and deformity) [20]. Based on the World Health Organization-Uppsala Monitoring Centre (WHO-UMC) criteria, ADE records with “certain”, “probable/likely”, and “possible” causality were included in the analysis [29]. All ADEs were reported in preferred terms or included terms, according to the WHO-Adverse Reaction Terminology (WHO-ART), and were further grouped into system organ class (SOC) disorders. Serious adverse events (SAEs) were classified as any AEs leading to death, life-threatening conditions, hospitalisation or prolongation of existing hospitalisation, persistent or significant disability or incapacity, congenital abnormalities or birth defects, and other medically significant events based on the International Conference on Harmonization (ICH) E2D guideline [30]. The pharmacovigilance study protocol utilising the KIDS-KD ADE records was approved by Korea Institute of Drug Safety & Risk Management (Ministry of Food and Drug Safety) (No.2007A0051) and the institutional review board of Kyung Hee University (No. KHSIRB-22-486) (Seoul, South Korea). Informed consents were exempted by the board.

### Statistical analysis

We estimated 3529 ADE reports would provide 80% power to demonstrate different SAE incidences among anti-obesity agents. We used descriptive statistics to summarise patient demographics and reported ADEs. We expressed continuous variables as medians (range; interquartile range (IQR)) based on the Kolmogorov-Smirnov normality test results. Disproportionality analysis, a primary quantitative ADE signal detection tool for RWD-based pharmacovigilance investigation to generate hypotheses on the possible causal relationship between ADEs and drugs, was performed to determine the association of the SOC-based ADEs with their seriousness and with sex by estimating reporting odds ratios (RORs) with their 95% confidence intervals (CIs) and Mantel-Haenszel adjusted *P*-values [31,32]. We subjected ADEs with at least four reported cases to the disproportionality analysis to ensure the reliability of the results [15,31]. We performed univariate analysis to identify factors associated with increased risk of SAEs, including sex, age, number and types of concurrently administered medications, causality (certain, probable/likely, or possible), and types of ADE reporters. We used a enter method for multiple logistic regression to determine which predictors are substantially associated with increased risk of SAEs from the univariate analysis. We examined clinical plausibility in the multiple logistic regression. We further performed subgroup analyses on reporter variability regarding association of SOC-based ADEs with their seriousness or sex using the disproportionality test, estimating the results with RORs and 95% CIs. We performed additional logistic regression to identify predictors substantially increasing risk of SAEs across the variable types of reporters, estimating their effects with ORs and corresponding 95% confidence interval. We used SPSS, version 26.0 (IBM SPSS Statistics for Windows, Armonk, New York, USA) for all statistical analyses. We considered *P-*values <0.05 as statistically significant.

## RESULTS

### Baseline demographics

**Figure 1:**
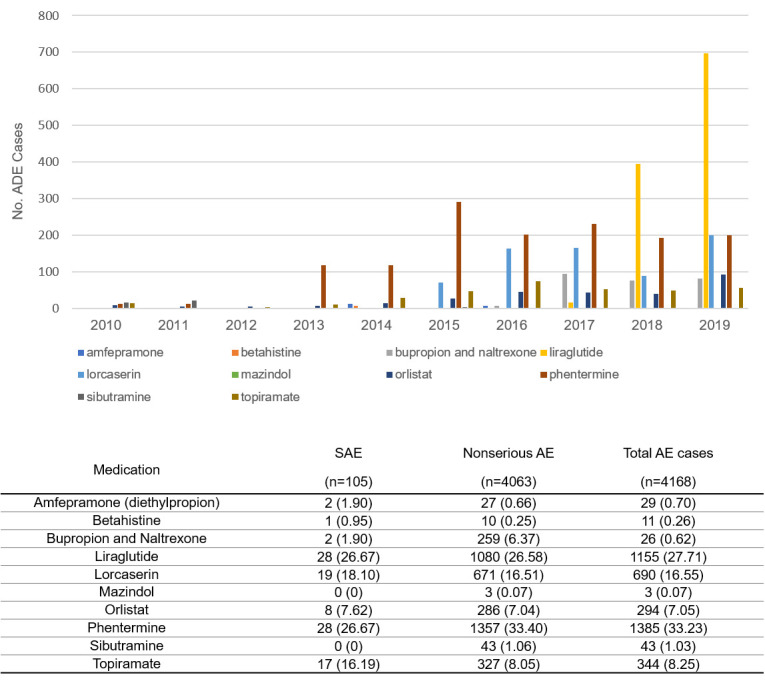
The number of ADE reports of each anti-obesity medication from 2010 to 2019.

Among 13 766 ADE records induced by obesity treatment reported to the KIDS-KD from January 2010 to December 2019, 4168 (30%) were identified as “certain”, “probable/likely”, or “possible” causality. The number of ADE reports has substantially increased, especially for liraglutide (**Figure 1**). Phentermine was the most common aetiologic agent (n = 1385 (33.23%)), followed by liraglutide (n = 1155 (27.71%)) and lorcaserin (n = 690 (16.55%)). Among 105 SAEs induced by anti-obesity medications, liraglutide and phentermine were responsible for most SAE cases (n = 28 (26.67%)), followed by lorcaserin (n = 19 (18.10%)) and topiramate (n = 17 (16.19%)). Most ADE cases were reported by women (n = 3430 (89.02%)). The median age of patients was 41 (IQR = 19) with most patients being 30 to 49 years old (n = 1194 (43.32%)). The most ADEs induced by obesity treatment were reported by the pharmacists (n = 1729 (44.62%)) and the general public (n = 1149 (29.63%)) (**Table 1**).

**Table 1:** Baseline demographic characteristic of patients from whom adverse drug events (ADEs) caused by weight loss medications

Characteristics	Number of cases (%)
Age in years, median (range; IQR)*	41 (12-84; 19)
*10 ~ 19*	45 (1.63)
*20 ~ 29*	438 (15.85)
*30 ~ 39*	756 (27.36)
*40 ~ 49*	656 (23.74)
*50 ~ 59*	585 (21.17)
*60 ~ 69*	224 (8.11)
*≥70*	59 (2.14)
Sex†	
*Men*	404 (10.98)
*Women*	3430 (89.02)
Causality	
*Certain*	108 (2.59)
*Probable/likely*	1571 (37.69)
*Possible*	2489 (59.72)
Seriousness	
*Serious adverse events*	105 (2.52)
*Nonserious adverse events*	4063 (97.48)
Reporting individuals‡	
*Doctors*	671 (17.32)
*Pharmacists*	1729 (44.62)
*Nurses*	203 (5.24)
*General public*	1148 (29.63)
*Others*	124 (3.2)
Number of concomitantly used medications	
*1*	3133 (75.17)
*2*	403 (9.67)
*3*	259 (6.21)
*4*	155 (3.71)
*≥5*	218 (5.23)
Number of comorbidities	
*1*	4144
*2*	23
*≥3*	4

IQR – interquartile range

*Missing in 1405 (33.7%) cases.

†Missing in 377 (9.04%) cases.

‡Missing in 293 (7.03%) cases.

### Types of ADEs

**Figure 2:**
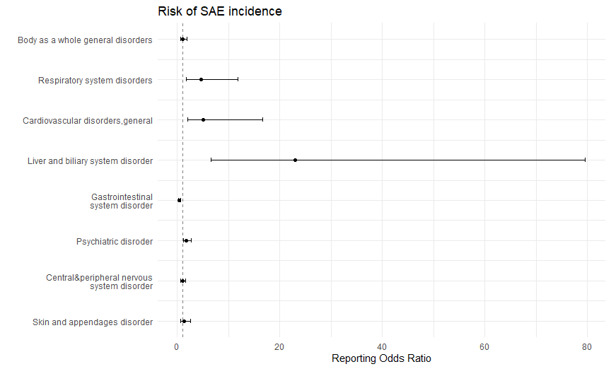
ADE reporting odds ratio on the association of seriousness with SOC-based ADEs.

**Figure 3:**
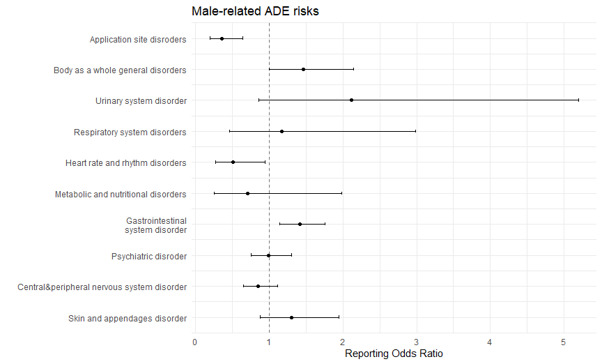
ADE reporting odds ratio on the association of SOC-based ADEs with sex.

The most common ADEs induced by anti-obesity medications were GI disorders (n = 1213 (29.10%)), central and peripheral nervous system disorders (n = 799 (19.17%)), and psychiatric disorders (n = 703 (16.87%)). The most frequently reported SAEs were associated with psychiatric disorders (n = 27 (25.71%)) and central and peripheral nervous system disorders (n = 20; 19.04%), with phentermine being the most aetiologic agent for psychiatric disorders (**Table 2**). ADEs associated with psychiatric disorders (ROR = 1.734; 95% CI = 1.111-2.707, *P* < 0.05), liver and biliary system disorders (ROR = 22.948; 95% CI = 6.613-70.635, *P* < 0.05), cardiovascular disorders (ROR = 5.707; 95% CI = 1.965-16.574, *P* = 0.002), and respiratory disorders (ROR = 4.567; 95% CI = 1.773-11.762, *P* < 0.05) were more likely to be serious events (**Figure 2**, Table S1 in the **Online Supplementary Document**). ADEs classified as GI disorders were least likely to be serious events (ROR = 0.337; 95% CI = 0.188-0.605, *P* < 0.05). Additionally, ADE risk related to GI system disorders (ROR = 1.411; 95% CI = 1.138-1.751, *P* < 0.05) and general disorders (ROR = 1.464; 95% CI = 1.002-2.139, *P* < 0.05) were higher in men, while ADE risk related to heart and rhythm disorders (ROR = 0.507; 95% CI = 0.274-0.941, *P* < 0.05) and application site disorders (ROR = 0.358; 95% CI = 0.199-0.644, *P* < 0.05) were higher in women (**Figure 3** and Table S2 in the **Online Supplementary Document**)

**Table 2:** System organ class-based adverse events caused by weight loss agents*

	Amfepramone (n = 29)	Betahistine (n = 11)	Bupropion and naltrexone (n = 261)	Liraglutide (n = 1155)	Lorcaserin (n = 690)	Mazindol (n = 3)	Orlistat (n = 294)	Phentermine (n = 1385)	Sibutramine (n = 43)	Topiramate (n = 344)
**Skin and appendages disorders**	1 (3.45)	2 (18.18)	9 (3.45)	116 (10.04)	34 (4.93)	0 (0)	16 (5.44)	77 (5.56)	2 (4.65)	13 (3.78)
**Musculo-skeletal system disorders**	0 (0)	0 (0)	3 (1.15)	5 (0.43)	37 (5.36)	0 (0)	2 (0.68)	7 (0.51)	0 (0)	4 (1.16)
**Central & peripheral nervous system disorders**	2 (6.90)	4 (36.36)	78 (29.89)	76 (6.58)	286 (41.45)	2 (66.67)	46 (15.65)	212 (15.31)	11 (25.58)	82 (23.84)
**Vision disorders**	0 (0)	0 (0)	2 (0.77)	1 (0.87)	4 (0.58)	0 (0)	3 (1.02)	8 (0.58)	0 (0)	7 (2.04)
**Hearing and vestibular disorders**	0 (0)	0 (0)	0 (0)	1 (0.87)	0 (0)	0 (0)	0 (0)	5 (0.36)	0 (0)	0 (0)
**Special senses other, disorders**	1 (3.45)	0 (0)	0 (0)	5 (0.43)	1 (0.14)	0 (0)	1 (0.34)	1 (0.07)	0 (0)	0 (0)
**Psychiatric disorders**	10 (34.48)	2 (18.18)	18 (6.90)	36 (3.12)	72 (10.43)	0 (0)	51 (17.35)	428 (30.90)	7 (16.27)	79 (22.97)
**Gastro-intestinal system disorders**	5 (17.24)	3 (27.27)	117 (44.83)	338 (29.26)	145 (21.01)	1 (33.33)	135 (45.92)	378 (27.29)	7 (16.27)	84 (24.42)
**Liver and biliary system disorders**	1 (3.45)	0 (0)	0 (0)	0 (0)	0 (0)	0 (0)	3 (1.02)	4 (0.29)	1 (2.33)	2 (0.58)
**Metabolic and nutritional disorders**	0 (0)	0 (0)	2 (0.77)	11 (0.95)	13 (1.88)	0 (0)	3 (1.02)	24 (1.73)	0 (0)	5 (1.45)
**Endocrine disorders**	0 (0)	0 (0)	0 (0)	0 (0)	0 (0)	0 (0)	0 (0)	2 (0.14)	0 (0)	0 (0)
**Cardiovascular disorders, general**	0 (0)	0 (0)	8 (3.07)	3(0.26)	1 (0.14)	0 (0)	0 (0)	16 (1.16)	0 (0)	4 (1.16)
**Heart rate and rhythm disorders**	6 (20.69)	0 (0)	7 (2.68)	10 (0.87)	18 (2.61)	0 (0)	7 (2.38)	129 (9.31)	5 (11.63)	20 (5.81)
**Vascular (extracardiac) disorders**	0 (0)	0 (0)	0 (0)	1 (0.09)	1 (0.14)	0 (0)	0 (0)	2 (0.14)	0 (0)	0 (0)
**Respiratory system disorders**	2 (6.90)	0 (0)	2 (0.77)	9 (0.78)	11 (1.59)	0 (0)	1 (0.34)	18 (1.30)	0 (0)	6 (1.74)
**Red blood cell disorders**	0 (0)	0 (0)	0 (0)	1 (0.09)	0 (0)	0 (0)	0 (0)	0 (0)	1 (2.33)	0 (0)
White cell and RES* disorders	0 (0)	0 (0)	0 (0)	0 (0)	0 (0)	0 (0)	0 (0)	0 (0)	0 (0)	1 (0.29)
**Platelet, bleeding & clotting disorders**	0 (0)	0 (0)	0 (0)	3 (0.26)	0 (0)	0 (0)	1 (0.34)	1 (0.07)	0 (0)	0 (0)
**Urinary system disorders**	0 (0)	0 (0)	0 (0)	9 (0.78)	5 (0.72)	0 (0)	4 (1.36)	9 (0.65)	0 (0)	6 (1.74)
**Reproductive disorders, female**	0 (0)	0 (0)	2 (0.77)	21 (1.82)	2 (0.29)	0 (0)	2 (0.68)	2 (0.14)	4 (9.30)	6 (1.74)
**Foetal disorders**	0 (0)	0 (0)	0 (0)	0 (0)	0 (0)	0 (0)	1 (0.34)	0 (0)	0 (0)	0 (0)
**Neoplasms**	0 (0)	0 (0)	0 (0)	2 (0.17)	0 (0)	0 (0)	0 (0)	1 (0.07)	0 (0)	0 (0)
**Body as a whole – general disorders**	1 (3.45)	0 (0)	12 (4.60)	339 (29.10)	58 (8.41)	0 (0)	17(5.78)	57 (4.12)	5 (11.63)	24 (6.98)
**Application site disorders**	0 (0)	0 (0)	0 (0)	100 (8.59)	1 (0.14)	0 (0)	0 (0)	0 (0)	0 (0)	0 (0)
**Secondary terms – events**	0 (0)	0 (0)	1 (0.38)	21 (1.80)	1 (0.14)	0 (0)	1 (0.34)	3 (0.22)	0 (0)	1 (0.29)
**Poison specific term**	0 (0)	0 (0)	0 (0)	0 (0)	0 (0)	0 (0)	0 (0)	1 (0.07)	0 (0)	0 (0)

*Values represented as n (%) unless otherwise specified.

### Predictors associated with increased risk SAE incidences

The univariate analysis found that sex, number and types of concomitantly used medications, and reporting personnel were predictors of increased risk of SAEs induced by anti-obesity medication (**Table 3**). The risk of SAE incidences was substantially greater in men (OR = 2.196; 95% CI = 1.296-3.721, *P* < 0.05) and individuals taking a higher number of concurrent medications (OR = 1.182; 95% CI = 1.009-1.385, *P* < 0.05). The seriousness of AEs was also positively correlated with dual (OR = 3.258) or triple anti-obesity therapy (OR = 8.226) and concomitant administration of fluoxetine (OR = 5.236; 95% CI = 2.218-12.365, *P* < 0.05), while concurrent levocarnitine administration was less likely to increase potentiality of SAEs (OR = 0.085; 95% CI = 0.011-0.686, *P* < 0.05). Furthermore, compared to the doctors, the general public were more likely to report SAEs induced by anti-obesity medication (OR = 3.490; 95% CI = 1.248-9.757, *P* < 0.05). Pharmacist, however, were less likely to report SAEs than doctors (OR = 0.069; 95% CI = 0.020-0.163).

**Table 3:** Univariate and multivariate analyses for the association between predictors and serious adverse events (SAEs)

	Univariate analysis, number of cases (%)*			Multivariate Analysis†	
**Predictors**	**SAEs (n = 105)**	**Non-serious AEs (n = 4063)**	***P-*value**	**OR (95% CI)**	***P-*value**
Sex			<0.05		<0.05
*Men*	7 (1.73)	397 (98.27)		2.196 (1.296-3.721)	
*Women*	30 (0.88)	3356 (99.11)		1 (reference)	
Age	>0.05				
*10-19*	2	43			
*20-29*	10	428			
*30-39*	21	735			
*40-49*	17	639			
*50-59*	15	570			
*60-69*	2	222			
*≥70*	2	57			
Causality			>0.05		
*Certain*	2 (1.85)	106 (98.15)			
*Probable/likely*	38 (2.42)	1533 (97.58)			
*Possible*	65 (2.61)	2424 (97.39)			
Number of concurrently used medications				1.182 (1.009-1.385)	<0.05
*1*	56 (1.78)	3077 (98.21)			
*2*	17 (4.22)	386 (95.78)			
*3*	16 (6.18)	243 (93.82)			
*4*	7 (4.52)	148 (95.48)			
*≥5*	9 (4.13)	209 (95.87)			
Types of concomitantly used medication					
Dual anti-obesity therapy	25 (4.71)	506 (95.29)	<0.05	3.258 (1.633-6.501)	<0.05
Triple anti-obesity therapy	12 (9.52)	114 (90.48)	<0.05	8.226 (3.046-22.218)	<0.05
Fluoxetine	19 (14.62)	111 (85.38)	<0.05	5.236 (2.218-12.365)	<0.05
Levocarnitine	1 (1.37)	72 (98.63)	<0.05	0.085 (0.011-0.686)	<0.05
Reporting personnel			<0.05		
*Doctors*	27 (4.02)	644 (95.98)		1 (reference)	
*Pharmacists*	8 (0.46)	1721 (99.54)		0.069 (0.030-0.163)	
*Nurses*	6 (2.96)	197 (97.04)		1.063 (0.412-2.744)	
*General Public*	39 (3.40)	1109 (96.60)		3.490(1.248-9.757)	
*Others*	13 (10.48)	111 (89.52)		1.526 (0.711-3.273)	

CI – confidence interval, OR – odds ratio, SAEs - serious adverse events, AEs – adverse events

*Univariate logistic regression was performed to identify predictors associated with increased risk of SAEs.

†Multivariate logistic regression was performed based on the identified predictors (*P*-value >0.05 from univariate analysis).

### Subgroup analysis on reporter variability

**Figure 4:**
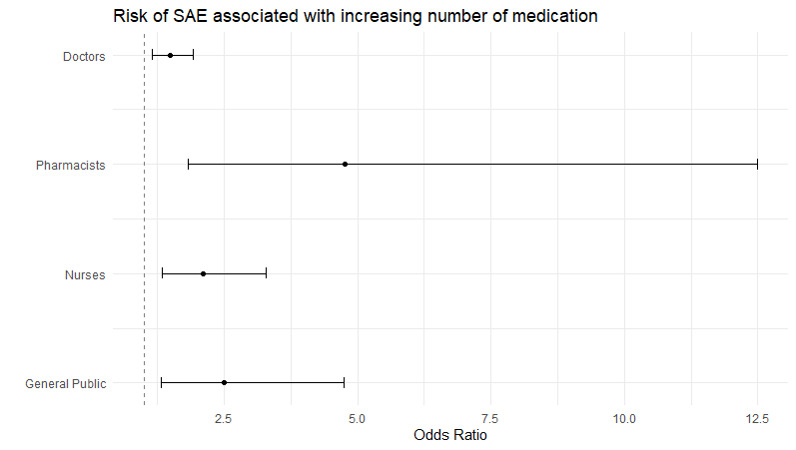
Subgroup analysis of reporter variability on the impact of increasing number of concomitant medications on SAE risk.

Among ADE records reported by the doctors, ADEs pertaining to skin ad appendages disorder (ROR = 3.172; 95% CI = 1.291-7.796, *P* < 0.05) were more likely to be SAEs, while ADEs related to general body as whole disorders were more likely to be SAEs in ADE records filed by the pharmacists (ROR = 9.994; 95% CI = 2.949-33.870, *P* < 0.05) (**Table 4**). The general public was more likely to report SAE cases related to central and peripheral nervous system disorders (ROR = 7.203; 95% CI = 3.045-17.041, *P* < 0.05) and general body as whole disorders (ROR = 3.207; 95% CI = 1.057-9.725, *P* < 0.05) (**Table 4**). Men had higher AE risk related to GI system disorders based on the ADE cases reported by the pharmacist (ROR = 1.689; 95% CI = 1.179-2.422, *P* < 0.05) while we found lower AE risk related to application site disorders in men in ADE cases reported by public (ROR = 0.345; 95% CI = 0.191-0.657, *P* < 0.05) (**Table 5**). The increased number of concomitant medications with anti-obesity medication was the most contributing predictor for elevating SAE risk across all reporters: doctors (OR = 1.487; 95% CI = 1.153-1.918, *P* < 0.05), pharmacist (OR = 4.759; 95% CI = 1.820-12.500, *P* < 0.05), nurses (OR = 2.110; 95% CI = 1.353-3.289, *P* < 0.05), and general public (OR = 2.503; 95% CI = 1.323-4.738, *P* < 0.05) (**Figure 4**, Tables S3-S12 in the **Online Supplementary Document**).

**Table 4:** Subgroup analysis of association between system Organ Class-based adverse events and seriousness of ADE classified by types of reporters*

	Serious AEs	Nonserious AEs	Total AEs	*P*-values	ROR (95% CI)
**Doctors**	**n = 105**	**n = 4063**	**n = 4168**		
Skin and appendages disorders	7 (25.9)	63 (9.8)	70 (10.4)	<0.05	3.172 (1.291-7.796)
Central & peripheral nervous system disorders	5 (18.5)	141 (21.9)	146 (21.8)	>0.05	0.811 (0.302-2.179)
Gastro-intestinal system disorders	5 (18.5)	203 (31.5)	208 (31.0)	>0.05	0.494 (0.184-1.322)
**Pharmacists**	**n = 12**	**n = 1721**	**n = 1733**		
Body as a whole – general disorders	4 (33.3)	82 (4.8)	86 (5.0)	<0.05	9.994 (2.949-33.870)
**General public**	**n = 22**	**n = 1109**	**n = 1131**		
Central & peripheral nervous system disorders	10 (45.5)	115 (10.4)	125 (11.1)	<0.05	7.203 (3.045-17.041)
Gastro-intestinal system disorders	5 (22.7)	307 (27.7)	312 (28.6)	>0.05	0.768 (0.281-2.101)
Body as a whole – general disorders	4 (18.2)	72 (6.5)	76 (6.7)	<0.05	3.207 (1.057-9.725)

AEs – adverse events, ROR – reporting odds ratios, CI – confidence interval

*Values represented as n (%) unless otherwise specified.

**Table 5:** Subgroup analysis on the association between System Organ Class-based adverse events and gender classified by type of reporters*

	AEs in male	AEs in female	Total AEs	*P*-values	ROR (95% CI)
**Doctor**	**n = 97**	**n = 527**	**n = 624**		
Skin and appendages disorders	9 (9.3)	50 (9.5)	59 (9.5)	>0.05	0.965 (0.458-2.032)
Central & peripheral nervous system disorders	19 (19.6)	121 (23.0)	140 (22.4)	>0.05	10.817 (0.476-1.404)
Psychiatric disorders	20 (20.6)	81 (15.4)	101 (16.2)	>0.05	1.430 (0.829-2.469)
Gastro-intestinal system disorders	36 (37.1)	163 (30.9)	199 (31.9)	>0.05	1.318 (0.839-2.070)
Body as a whole - general disorders	6 (6.2)	37 (7.0)	43 (6.9)	>0.05	0.873 (0.358-2.129)
**Pharmacists**	**n = 140**	**n = 1502**	**n = 1642**		
Skin and appendages disorders	9 (6.4)	59 (3.9)	68 (4.1)	>0.05	1.680 (0.815-3.465)
Central & peripheral nervous system disorders	28 (20)	335 (22.3)	363 (22.1)	>0.05	0.871 (0.566-1.341)
Psychiatric disorders	28 (20)	385 (25.6)	413 (25.2)	>0.05	0.725 (0.472-1.115)
Gastro-intestinal system disorders	54 (38.6)	414 (27.6)	468 (28.5)	<0.05	1.689 (1.179-2.422)
Respiratory system disorders	4 (2.9)	13 (0.9)	17 (1.0)	<0.05	3.369 (1.084-10.474)
Body as a whole – general disorders	5 (3.6)	72 (4.8)	77 (4.7)	>0.05	0.736 (0.292-1.852)
**Nurses**	**n = 33**	**n = 270**	**n = 203**		
Psychiatric disorders	7 (21.2%)	19 (11.2%)	26 (12.8%)	>0.05	2.140 (0.818-5.596)
Gastro-intestinal system disorders	14 (42.4%)	65 (38.2%)	79 (38.9%)	>0.05	1.190 (0.559-2.536)
**General public**	**n = 95**	**n = 930**	**n = 1025**		
Central & peripheral nervous system disorders	14 (14.7%)	99 (10.6%)	113 (11.0%)	>0.05	1.451 (0.793-2.655)
Psychiatric disorders	9 (9.5%)	75 (8.1%)	84 (8.2%)	>0.05	1.193 (0.577-2.466)
Gastro-intestinal system disorders	36 (37.9%)	273 (29.4%)	309 (30.1%)	>0.05	1.468 (0.085-2.275)
Body as a whole – general disorders	11 (11.6%)	65 (7.0%)	76 (7.4%)	>0.05	1.746 (0.885-3.430)
Application site disorders	11 (11.6%)	256 (27.5%)	267 (26.0%)	<0.05	0.345 (0.191-0.657)
**Others**	**n = 13**	**n = 48**	**n = 61**		
Psychiatric disorders	6 (46.2)	8 (16.7)	14 (23.0)	<0.05	4.286 (1.135-16.182)

AEs – adverse events, CI – confidence interval, ROR – reporting odds ratio

*Values presented as n (%) unless otherwise specified.

## DISCUSSION

Most ADE reports pertaining to anti-obesity medications were collected from women aged 20 to 50, with over 70% of patients taking only one medication. The incidence of SAEs induced by anti-obesity medications was 2.52%, with phentermine being the most commonly identified causative agent, followed by liraglutide. While the most ADE cases were associated with GI disorders, ADEs associated with psychiatric disorders, liver and biliary system disorders, and respiratory disorders were more likely to lead to serious events. Furthermore, we found sex differences in ADE profiles, with a higher incidence of GI-related ADEs in men and a higher incidence of heart and rhythm disorders in women. The multivariate analysis revealed that being male, taking an increasing number of medications, multiple administration of anti-obesity medications, and concomitant administration of fluoxetine are predictors associated with significantly increased risk of SAE incidences.

The greatest benefit of utilising RWD-derived spontaneous ADE reports in pharmacovigilance investigation is providing evidence representative of real-world clinical practice, as evidenced by the correlation between high numbers of ADE cases and the large number of prescriptions. For example, phentermine has been the most prescribed anti-obesity agent globally, including in South Korea [33,34]. However, liraglutide (Saxenda®), the first glucagon-like peptide 1 (GLP-1) analogue for weight loss, was approved in 2017 by the South Korean Ministry of Food and Drug Safety [35]. It has gained significant popularity due to its substantial weight reduction along with favourable safety profiles, resulting in an increase in both ADE reports and prescriptions [36]. Another example of real-word representation is the considerably higher ADE prevalence in women. Nearly 90% of ADE reports were collected from women, and more than half of patients were aged between 20 to 50. Studies have demonstrated women usually have higher drug utilisation and adherence rate compared to men [37-39]. Furthermore, evidence suggest relatively lower prescription rate of anti-obesity medication in men, despite them having similar obesity rate to women [40]. Moreover, in line with our findings, younger females, typically aged between 35-54 years, are more likely to be prescribed anti-obesity medications than older aged women [40].

Our findings related to personnel involved in ADE reporting are unexpected. Traditionally, health care professionals such as physicians, pharmacists, and nurses have been the primary reporters of ADE cases in pharmacovigilance studies [15,41]. However, the proportion of ADE reports collected from the general public is relatively higher than other pharmacovigilance studies, accounting for 29.63% of total ADE cases [15,41]. Among 1148 ADE records reported by the general public in our study, 870 cases were specifically related to liraglutide-induced ADEs, mostly related to non-serious GI system disorders, highlighting the popularity of liraglutide among the public in Korea. However, based on the results of the subgroup analysis on the impact of reporter variability on the association of SOC-based ADEs with seriousness, central and peripheral nervous system disorders and general body disorders are more likely to be SAEs among ADE records reported by general public. The European Medicines Agency recently initiated a pharmacovigilance investigation of GLP-1 agonist prescribed for obesity management for the risk of suicidal thoughts [42]. Such comprehensive pharmacovigilance investigation based on extensive data collection and analysis, along with increasing ADE awareness in the public, are key to ensuring patient safety. Notable, the multivariate analysis in our study showed that pharmacists are less likely to report serious ADEs than doctors; this could be attributed to differences in clinical practice settings, as pharmacists are more likely to encounter patients experiencing non-serious ADEs than doctors. This finding could also have resulted from the relatively large number of ADE cases reported by pharmacist, considering their professional obligation to detect ADE signals [43].

A major safety concern in obesity treatment is concomitant administration of medications, including multiple anti-obesity medications. Although most patients received only one weight loss medications, about 16% administered dual or triple anti-obesity medication; according to our multivariate analysis, such concomitant administration substantially elevates the risk of SAEs. Moreover, subgroup analysis of reporter-variability identified an increasing number of concomitant medications as the primary predictor for increasing SAE risk induced by anti-obesity medications, and some patients were concurrently taking other agents with potential weight loss properties, such as fluoxetine and levocarnitine. The multivariate analysis revealed markedly elevated risk of SAEs with concomitant administration of fluoxetine and anti-obesity agents. Although fluoxetine is not classified as anti-obesity agents, studies revealed modest weight reduction of 2.7kg with fluoxetine administration; thus, it is possible that some patients may be using fluoxetine for weight loss [44]. However, considering that obese populations are at elevated risk of depression, concomitant administration of fluoxetine may be more common than reported ADE cases. Meanwhile, a recent meta-analysis demonstrated modest weight reduction of 1.21kg with levocarnitine, and some patients may administer levocarnitine supplementation as adjunctive obesity treatment to accelerate weight loss. Although the multivariate analysis revealed relatively lower OR of SAE incidence, caution should be exercised when managing obese patients due to potential drug-interactions [45]. Additionally, considering the elevated risk of chronic complications including T2DM, hypertension, cardiovascular diseases, and cancer [4], obese populations are predisposed to the risk of taking large number of medications, consequently increasing susceptibility for increased SAE risk. Thus, further controlled clinical studies evaluating the risks associated with concomitant administration of anti-obesity agents and other medications including antidepressants and medications for comorbid diseases are warranted to determine the absolute risk of ADEs from drug interactions.

The primary rationale for post-market withdrawals is medication safety, mostly articulated as adverse events. Post-market withdrawals seem common with anti-obesity medications, as evidenced by 25 post-market withdrawal cases reported between 1964 and 2009 [13]. Additional medication withdrawal has been reported during our study period. Sibutramine, for example, was withdrawn from market by European Medicine Agency (EMA) in 2010 due to increased risk of heart attack and stroke in patients with high cardiovascular risk [35]. Subsequently, sibutramine was also withdrawn in South Korea [46]. Furthermore, locarserin and amfepramone were withdrawn by the FDA and the EMA [47-49]. The primary reason lorcaserin was primarily withdrawn were the increased risks of cancer incidences, while amfepramone was withdrawn due to inappropriate use, particularly when prescribed outside the risk minimisation measures suggested by the EMA [49]. Inappropriate use of amfepramone can induce serious AEs including elevation of blood pressure in arteries of lungs, CVDs, dependency and psychiatric problems [49]. Although we were unable to determine the correlation between amfepramone dose and the incidence and seriousness of ADEs, the most reported amfepramone-induced ADEs include psychiatric disorders and heart rate and rhythm disorders. Other anti-obesity agents on market, such as liraglutide, may be considered as relatively safe than withdrawn agents, however, cautious monitoring is required, regardless of type of anti-obesity agents, as the increased risk of serious psychiatric and liver and biliary system disorder were observed with these medications.

Despite benefits of using RWD for pharmacovigilance investigation, our study has several limitations. First, as KIDS-KD is a spontaneous voluntary ADE reporting system, our findings should be interpreted with caution due to potential under-reporting AEs in addition to possible omission of significant information, such as comorbid conditions, indications, and concurrent medication therapy. Additionally, the quality of collected reports might be influenced by inter-reporter variability. As we have discussed previously, nearly 1/3 of ADE reports were collected from general public, otherwise referred to as consumers. As consumers tentatively have less awareness and knowledge on pharmacotherapy-induced ADEs, potential reporting bias might exist. Also, the seriousness of the reported ADEs might be based on subjective judgment. Nevertheless, there should be minimal bias from ADE cases reported by the public, as the South Korea Institute of Drug Safety & Risk Management (Ministry of Food & Drug Safety) performs further in-depth investigation by reviewing patient’s medical charts, interviewing patient and health care professionals, and collecting scientific pharmacovigilance data from manufacturers prior to causality assessment and data storage to KIDS-KD. Moreover, any ADE cases reported by public undergoes preceding review process by reginal pharmacovigilance centers designated by Ministry of Food & Drug Safety or the manufacturers prior to KIDS investigation. Among 1148 ADE cases reported by the general public, 978 and 170 cases were indeed verified by drug manufacturers and regional pharmacovigilance centres, respectively, implying adequate reliability and appropriateness of the data sources. Lastly, we could note establish causality between the reported ADEs and the offending agents due to the cross-sectional study design. Moreover, although we obtained sufficient sample size based on the sample size calculation, cautious interpretation is required when generalising our results, as some statistical analyses, including the subgroup analysis, were performed with small sample size, subsequently providing large ranges of 95% CI estimates. Hence, further large-scaled clinical studies, along with ongoing data collection and analysis, are key to determining the impact of a specific anti-obesity medication, including newly approved agent, on the risk of the reported ADEs in consideration of potential confounding variables such as age, sex, comorbid disease, concomitant medications, and the perspectives of ADE reporters.

## CONCLUSION

The prevalence of ADE cases induced by anti-obesity medications has been increasing annually. In our study, the most aetiologic agent was phentermine, followed by liraglutide. The most common nonserious ADE cases were pertaining to GI related disorders such as vomiting and nausea, followed by psychiatric disorders. However, the most common serious ADEs were associated with psychiatric disorders and central and peripheral nervous system. Variable ADE profiles are observed with sex; men are more likely to be reported with GI-related ADEs whereas women are at elevated risk of reporting ADEs associated with heart rate and rhythm disorders. The predictors associated with substantial increase in SAEs of anti-obesity medication were sex, number and types of concomitantly used medications, and ADE reporting personnel. Nevertheless, ongoing ADE collection and future analysis on anti-obesity medications is imperative, considering limited RWD-based pharmacovigilance investigation along with the limitations of this study. Moreover, further studies are needed to determine the impact of a specific anti-obesity medication, including newly approved agent, on the risk of the reported ADEs in consideration of potential confounding variables including age, sex, comorbid conditions, concomitant medications, and perspectives of ADE reporters. Moreover, close monitoring of ADE incidences pertaining to anti-obesity medications is crucial for promoting patient safety.

## Additional material


Online Supplementary Document
