# Supplementary material for: A nationwide pharmacovigilance investigation on trends and seriousness of adverse events induced by anti-obesity medication
Source: J Glob Health. 2023 Sep 1;13:04095. doi: 10.7189/jogh.13.04095 (PMC10471157; doi:10.7189/jogh.13.04095)
Supplement: Online Supplementary Document [file jogh-13-04095-s001.pdf]

Table S1. Association between system Organ Class-based adverse events and seriousness

	Serious AEs (n=105)	Nonserious AEs (n=4063)	Total AEs (n=4168)	*P*-values	ROR (95% CI)
**Skin and appendages disorders**	9 (8.57%)	261 (6.42%)	270 (6.48 %)	>0.05	1.249 (0.625-2.499)
**Musculo-skeletal system disorders**	1 (0.95%)	57 (1.40%)	58 (1.39%)	N/E	N/E
**Central & peripheral nervous system disorders**	20 (19.05%)	779 (19.17%)	799 (19.17%)	>0.05	0.992 (0.606-1.625)
**Vision disorders**	2 (1.90%)	23 (0.57%)	25 (0.60%)	N/E	N/E
**Hearing and vestibular disorders**	0 (0%)	6 (0.15%)	6 (0.14%)	N/E	N/E
**Special senses other, disorders**	0 (0%)	9 (0.22%)	9 (0.22%)	N/E	N/E
**Psychiatric disorders**	27 (25.71%)	676 (16.64%)	703 (16.87%)	<0.05	**1.734 (1.111-2.707)**
**Gastro-intestinal system disorders**	13 (12.38%)	1200 (29.53%)	1213 (29.10%)	<0.05	**0.337 (0.188-0.605)**
**Liver and biliary system disorders**	4 (3.81%)	7 (0.17%)	11 (0.26%)	<0.05	**22.948 (6.613-70.635)**
**Metabolic and nutritional disorders**	2 (1.09%)	56 (1.38%)	58 (1.39%)	N/E	N/E
**Endocrine disorders**	0 (0%)	2 (0.05%)	2 (0.05%)	N/E	N/E
**Cardiovascular disorders, general**	4 (3.81%)	28 (0.69%)	32 (0.77%)	<0.05	**5.707 (1.965-16.574)**
**Heart rate and rhythm disorders**	0 (0%)	202 (4.97%)	202 (4.84%)	N/E	N/E
**Vascular (extracardiac) disorders**	1 (0.95%)	3 (0.07%)	4 (0.10%)	N/E	N/E
**Respiratory system disorders**	5 (4.76%)	44 (1.08%)	49 (1.18%)	<0.05	**4.567 (1.773-11.762)**
**Red blood cell disorders**	0 (0%)	2 (0.05%)	2 (0.05%)	N/E	N/E
**White cell and RES* disorders**	0 (0%)	1 (0.02%)	1 (0.02%)	N/E	N/E
**Platelet, bleeding & clotting disorders**	0 (0%)	5 (0.12%)	5 (0.12%)	N/E	N/E
**Urinary system disorders**	0 (0%)	33 (0.81%)	33 (079%)	N/E	N/E
**Reproductive disorders, female**	0 (0%)	39 (0.95%)	39 (0.94%)	N/E	N/E
**Foetal disorders**	0 (0%)	1 (0.02%)	1 (0.02%)	N/E	N/E
**Neoplasms**	0 (0%)	3 (0.07%)	3 (0.07%)	N/E	N/E
**Body as a whole - general disorders**	13 (12.38%)	500 (12.31%)	513 (12.31%)	>0.05	1.007 (0.559-1.813)
**Application site disorders**	1 (0.95%)	100 (2.46%)	101 (2.42%)	N/E	N/E
**Secondary terms - events**	3 (2.86%)	25 (0.62%)	28 (0.67%)	N/E	N/E
**Poison specific terms**	0 (0%)	1 (0.02%)	1 (0.02%)	N/E	N/E

Table S2. Association between System Organ Class-based adverse events and gender

	AEs in Male (n=404)	AEs in Female (n=3386)	Total AEs (n=4168)	*P*-values	ROR (95% CI)
**Skin and appendages disorders**	30 (7.43%)	196 (5.79%)	226 (5.96%)	>0.05	1.306 (0.876-1.945)
**Musculo-skeletal system disorders**	3 (0.74%)	52 (1.54%)	55 (1.45%)	N/E	N/E
**Central & peripheral nervous system disorders**	71 (17.57%)	682 (20.14%)	753 (19.87%)	>0.05	0.848 (0.647-1.110)
**Vision disorders**	3 (0.74%)	21 (0.62%)	24 (0.63%)	N/E	N/E
**Hearing and vestibular disorders**	0 (0%)	6 (0.18%)	6 (0.16%)	N/E	N/E
**Special senses other, disorders**	1 (0.25%)	8 (0.24%)	9 (0.24%)	N/E	N/E
**Psychiatric disorders**	69 (17.08%)	582 (17.19%)	651 (17.18%)	>0.05	0.992 (0.754-1.305)
**Gastro-intestinal system disorders**	148 (36.63%)	984 (29.06%)	1132 (29.87%)	<0.05	**1.411 (1.138-1.751)**
**Liver and biliary system disorders**	1 (0.25%)	7 (0.21%)	8 (0.21%)	N/E	N/E
**Metabolic and nutritional disorders**	4 (0.99%)	47 (1.39%)	51 (1.35%)	0.669	0.710 (0.255-1.982)
**Endocrine disorders**	0 (0%)	2 (0.06%)	2 (0.05%)	N/E	N/E
**Cardiovascular disorders, general**	1 (0.25%)	27 (0.80%)	28 (0.74%)	N/E	N/E
**Heart rate and rhythm disorders**	11 (2.72%)	177 (5.23%)	188 (4.96%)	<0.05	**0.507 (0.274-0.941)**
**Vascular (extracardiac) disorders**	0 (0%)	4 (0.12%)	4 (0.11%)	N/E	N/E
**Respiratory system disorders**	5 (1.24%)	36 (1.06%)	41 (1.08%)	>0.05	1.166 (0.455-2.989)
**Red blood cell disorders**	1 (0.24%)	1 (0.03%)	2 (0.05%)	N/E	N/E
**White cell and RES* disorders**	1 (0.24%)	0 (0%)	1 (0.03%)	N/E	N/E
**Platelet, bleeding & clotting disorders**	0 (0 %)	4 (0.12%)	4 (0.11%)	N/E	N/E
**Urinary system disorders**	6 (1.49%)	24 (0.71%)	30 (0.79%)	>0.05	2.112 (0.858–5.197)
**Reproductive disorders, female**	0 (0%)	36 (1.06%)	36 (0.95%)	N/E	N/E
**Foetal disorders**	0 (0%)	1 (0.03%)	1 (0.03%)	N/E	N/E
**Neoplasms**	1 (0.25%)	1 (0.03%)	2 (0.05)	N/E	N/E
**Body as a whole - general disorders**	34 (8.42%)	200 (5.91%)	234 (6.17%)	<0.05	**1.464 (1.002-2.139)**
**Application site disorders**	12 (2.97%)	267 (7.89%)	279 (7.36%)	<0.05	**0.358 (0.199-0.644)**
**Secondary terms - events**	1 (0.25%)	21 (0.62%)	22 (0.58%)	N/E	N/E
**Poison specific terms**	1 (0.25%)	0 (0%)	1 (0.03%)	N/E	N/E

Table S3. Association between system Organ Class-based adverse events and seriousness of ADE reported by the doctors

	Serious AEs (n=27)	Nonserious AEs (n=644)	Total AEs (n=671)	*P*-values	ROR (95% CI)
**Skin and appendages disorders**	7 (25.9%)	63 (9.8%)	70 (10.4%)	**<0.05**	**3.172 (1.291-7.796)**
**Musculo-skeletal system disorders**	0 (0%)	1 (0.2%)	1 (0.1%)	N/E	N/E
**Central & peripheral nervous system disorders**	5 (18.5%)	141 (21.9%)	146 (21.8%)	>0.05	0.811 (0.302-2.179)
**Vision disorders**	0 (0%)	2 (0.2%)	2 (0.3%)	N/E	N/E
**Hearing and vestibular disorders**	0 (0%)	3 (0.5%)	3 3 (0.4%)	N/E	N/E
**Special senses other, disorders**	0 (0%)	0 (0%)	0 (0%)	N/E	N/E
**Psychiatric disorders**	3 (11.1%)	99 (15.4%)	102 (15.2%)	N/E	N/E
**Gastro-intestinal system disorders**	5 (18.5%)	203 (31.5%)	208 (31.0%)	>0.05	0.494 (0.184-1.322)
**Liver and biliary system disorders**	3 (11.1%)	1 (0.2%)	4 (0.6%)	N/E	N/E
**Metabolic and nutritional disorders**	0 (0%)	5 (0.8%)	5 (0.7%)	N/E	N/E
**Endocrine disorders**	0 (0%)	0 (0%)	0 (0%)	N/E	N/E
**Cardiovascular disorders, general**	1 (3.7%)	8 (1.2%)	9 (1.3%)	N/E	N/E
**Heart rate and rhythm disorders**	0 (0%)	31 (4.8%)	31 (4.6%)	N/E	N/E
**Vascular (extracardiac) disorders**	0 (0%)	3 (0.5%)	3 3 (0.4%)	N/E	N/E
**Respiratory system disorders**	3 (11.1%)	6 (0.9%)	9 (1.3%)	N/E	N/E
**Red blood cell disorders**	0 (0%)	0 (0%)	0 (0%)	N/E	N/E
**White cell and RES* disorders**	0 (0%)	1 (0.2%)	1 (0.1%)	N/E	N/E
**Platelet, bleeding & clotting disorders**	0 (0%)	1 (0.2%)	1 (0.1%)	N/E	N/E
**Urinary system disorders**	0 (0%)	3 (0.5%)	3 (0.4%)	N/E	N/E
**Reproductive disorders, female**	0 (0%)	0 (0%)	0 (0%)	N/E	N/E
**Foetal disorders**	0 (0%)	5 (0.7%)	5 (0.7%)	N/E	N/E
**Neoplasms**	0 (0%)	0 (0%)	0 (0%)	N/E	N/E
**Body as a whole - general disorders**	0 (0%)	45 (6.9%)	45 (6.7%)	N/E	N/E
**Application site disorders**	0 (0%)	20 (3.1%)	20 (3.0%)	N/E	N/E
**Secondary terms - events**	0 (0%)	2 (0.3%)	2 (0.3%)	N/E	N/E
**Poison specific terms**	0 (0%)	1 (0.2%)	1 (0.1%)	N/E	N/E

Table S4. Association between System Organ Class-based adverse events and gender reported by the doctors

	AEs in Male (n=97)	AEs in Female (n=527)	Total AEs (n=624)	*P*-values	ROR (95% CI)
**Skin and appendages disorders**	9 (9.3%)	50 (9.5%)	59 (9.5%)	>0.05	0.965 (0.458-2.032)
**Musculo-skeletal system disorders**	0 (0%)	1 (0.2%)	1 (0.2%)	N/E	N/E
**Central & peripheral nervous system disorders**	19 (19.6%)	121 (23.0%)	140 (22.4%)	>0.05	1.0.817 (0.476-1.404)
**Vision disorders**	0 (0%)	2 (0.4%)	2 (0.3%)	N/E	N/E
**Hearing and vestibular disorders**	0 (0%)	3 (0.6%)	3 (0.5%)	N/E	N/E
**Special senses other, disorders**	0 (0%)	0 (0%)	0 (0%)	N/E	N/E
**Psychiatric disorders**	20 (20.6%)	81 (15.4%)	101 (16.2%)	>0.05	1.430 (0.829 – 2.469)
**Gastro-intestinal system disorders**	36 (37.1%)	163 (30.9%)	199 (31.9%)	>0.05	1.318 (0.839-2.070)
**Liver and biliary system disorders**	0 (0%)	3 (0.6%)	3 (0.5%)	N/E	N/E
**Metabolic and nutritional disorders**	0 (0%)	5 (0.9%)	5 (0.9%)	N/E	N/E
**Endocrine disorders**	0 (0%)	0 (0%)	0 (0%)	N/E	N/E
**Cardiovascular disorders, general**	0 (0%)	5 (0.9%)	5 (0.9%)	N/E	N/E
**Heart rate and rhythm disorders**	2 (2.1%)	32 (6.1%)	34 (5.4%)	N/E	N/E
**Vascular (extracardiac) disorders**	0 (0%)	1 (0.2%)	1 (0.2%)	N/E	N/E
**Respiratory system disorders**	1 (1.0%)	10 (1.9%)	11 (1.8%)	N/E	N/E
**Red blood cell disorders**	0 (0%)	0 (0%)	0 (0%)	N/E	N/E
**White cell and RES* disorders**	1 (1.0%)	0 (0%)	1 (0.2%)	N/E	N/E
**Platelet, bleeding & clotting disorders**	0 (0%)	0 (0%)	0 (0%)	N/E	N/E
**Urinary system disorders**	1 (1.0%)	1 (0.2%)	2 (0.3%)	N/E	N/E
**Reproductive disorders, female**	0 (0%)	5 (0.9%)	5 (0.9%)	N/E	N/E
**Foetal disorders**	0 (0%)	0 (0%)	0 (0%)	N/E	N/E
**Neoplasms**	0 (0%)	0 (0%)	0 (0%)	N/E	N/E
**Body as a whole - general disorders**	6 (6.2%)	37 (7.0%)	43 (6.9%)	>0.05	0.873 (0.358-2.129)
**Application site disorders**	1 (1.0%)	7 (1.3%)	8 (1.3%)	N/E	N/E
**Secondary terms - events**	0 (0%)	0 (0%)	0 (0%)	N/E	N/E
**Poison specific terms**	1 (1.0%)	0 (0%)	1 (0.2%)	N/E	N/E

Table S5. Association between system Organ Class-based adverse events and seriousness of ADE reported by the pharmacists

	Serious AEs (n=12)	Nonserious AEs (n=1721)	Total AEs (n=1733)	*P*-values	ROR (95% CI)
**Skin and appendages disorders**	1 (8.3%)	69 (4.0%)	70 (4.0%)	N/E	N/E
**Musculo-skeletal system disorders**	0 (0%)	32 (1.9%)	32 (1.8%)	N/E	N/E
**Central & peripheral nervous system disorders**	2 (16.7%)	381 (22.1%)	383 (22.1%)	N/E	N/E
**Vision disorders**	0 (0%)	13 (0.8%)	13 (0.8%)	N/E	N/E
**Hearing and vestibular disorders**	0 (0%)	1 (0.1%)	1 (0.1%)	N/E	N/E
**Special senses other, disorders**	0 (0%)	1 (0.1%)	1 (0.1%)	N/E	N/E
**Psychiatric disorders**	2 (16.7%)	429 (24.9%)	431 (24.9%)	N/E	N/E
**Gastro-intestinal system disorders**	0 (0%)	493 (28.6%)	493 (28.4%)	N/E	N/E
**Liver and biliary system disorders**	1 (8.3%)	5 (0.3%)	6 (0.3%)	N/E	N/E
**Metabolic and nutritional disorders**	0 (0%)	23 (1.3%)	23 (1.3%)	N/E	N/E
**Endocrine disorders**	0 (0%)	0 (0%)	0 (0%)	N/E	N/E
**Cardiovascular disorders, general**	0 (0%)	19 (1.1%)	19 (1.1%)	N/E	N/E
**Heart rate and rhythm disorders**	0 (0%)	122 (7.1%)	122 (7.0%)	N/E	N/E
**Vascular (extracardiac) disorders**	0 (0%)	1 (0.1%)	1 (0.1%)	N/E	N/E
**Respiratory system disorders**	2 (16.7%)	17 (1.0%)	19 (1.1%)	N/E	N/E
**Red blood cell disorders**	0 (0%)	0 (0%)	0 (0%)	N/E	N/E
**White cell and RES* disorders**	0 (0%)	0 (0%)	0 (0%)	N/E	N/E
**Platelet, bleeding & clotting disorders**	0 (0%)	1 (0.1%)	1 (0.1%)	N/E	N/E
**Urinary system disorders**	0 (0%)	13 (0.8%)	13 (0.8%)	N/E	N/E
**Reproductive disorders, female**	0 (0%)	13 (0.8%)	13 (0.8%)	N/E	N/E
**Foetal disorders**	0 (0%)	0 (0%)	0 (0%)	N/E	N/E
**Neoplasms**	0 (0%)	2 (0.1%)	2 (0.1%)	N/E	N/E
**Body as a whole - general disorders**	4 (33.3%)	82 (4.8%)	86 (5.0%)	**<0.05**	**9.994 (2.949 – 33.870)**
**Application site disorders**	0 (0%)	0 (0%)	0 (0%)	N/E	N/E
**Secondary terms - events**	0 (0%)	3 (0.2%)	3 (0.2%)	N/E	N/E
**Poison specific terms**	0 (0%)	0 (0%)	0 (0%)	N/E	N/E

Table S6. Association between System Organ Class-based adverse events and gender reported by the pharmacists

	AEs in Male (n=140)	AEs in Female (n=1502)	Total AEs (n=1642)	*P*-values	ROR (95% CI)
**Skin and appendages disorders**	9 (6.4%)	59 (3.9%)	68 (4.1%)	>0.05	1.680 (0.815-3.465)
**Musculo-skeletal system disorders**	2 (1.4%)	29 (1.9%)	31 (1.9%)	N/E	N/E
**Central & peripheral nervous system disorders**	28 (20%)	335 (22.3%)	363 (22.1)	>0.05	0.871 (0.566 – 1.341)
**Vision disorders**	1 (0.1%)	12 (0.8%)	13 (0.8%)	N/E	N/E
**Hearing and vestibular disorders**	0 (0%)	0 (0%)	0 (0%)	N/E	N/E
**Special senses other, disorders**	0 (0%)	2 (0.1%)	2 (0.1%)	N/E	N/E
**Psychiatric disorders**	28 (20%)	385 (25.6%)	413 (25.2%)	>0.05	-/725 (0.472-1.115)
**Gastro-intestinal system disorders**	54 (38.6%)	414 (27.6%)	468 (28.5%)	**<0.05**	**1.689 (1.179 0 2.422)**
**Liver and biliary system disorders**	1 (0.1%)	4 (0.3%)	5 (0.3%)	N/E	N/E
**Metabolic and nutritional disorders**	1 (0.1%)	22 (1.5%)	23 (1.4%)	N/E	N/E
**Endocrine disorders**	0 (0%)	0 (0%)	0 (0%)	N/E	N/E
**Cardiovascular disorders, general**	0 (0%)	18 (1.2%)	18 (1.1%)	N/E	N/E
**Heart rate and rhythm disorders**	3 (2.1%)	111 (7.4%)	114 (6.9%)	N/E	N/E
**Vascular (extracardiac) disorders**	0 (0%)	1 (0.1%)	1 (0.1%)	N/E	N/E
**Respiratory system disorders**	4 (2.9%)	13 (0.9%)	17 (1.0%)	**<0.05**	3.369 (1.084 – 10.474)
**Red blood cell disorders**	0 (0%)	0 (0%)	0 (0%)	N/E	N/E
**White cell and RES* disorders**	0 (0%)	0 (0%)	0 (0%)	N/E	N/E
**Platelet, bleeding & clotting disorders**	0 (0%)	1 (0.1%)	1 (0.1%)	N/E	N/E
**Urinary system disorders**	3 (2.1%)	9 (-.6%)	12 (0.7%)	N/E	N/E
**Reproductive disorders, female**	0 (0%)	11 (0.7%)	11 (0.7%)	N/E	N/E
**Foetal disorders**	0 (0%)	0 (0%)	0 (0%)	N/E	N/E
**Neoplasms**	0 (0%)	0 (0%)	0 (0%)	N/E	N/E
**Body as a whole - general disorders**	5 (3.6%)	72 (4.8%)	77 (4.7%)	>0.05	0.736 (0.292-1.852)
**Application site disorders**	0 (0%)	2 (0.1%)	2 (0.1%)	N/E	N/E
**Secondary terms - events**	1 (0.1%)	2 (0.1%)	3 (0.2%)	N/E	N/E
**Poison specific terms**	0 (0%)	0 (0%)	0 (0%)	N/E	N/E

Table S7. Association between system Organ Class-based adverse events and seriousness of ADE reported by the nurses

	Serious AEs (n=6)	Nonserious AEs (n=197)	Total AEs (n=203)	*P*-values	ROR (95% CI)
**Skin and appendages disorders**	0 (0%)	21 (10.7%)	21 (10.3%)	N/E	N/E
**Musculo-skeletal system disorders**	0 (0%)	5 (2.5%)	5 (2.5%)	N/E	N/E
**Central & peripheral nervous system disorders**	0 (0%)	35 (17.8%)	35 (17.2%)	N/E	N/E
**Vision disorders**	0 (0%)	2 (1.0%)	2 (1.0%)	N/E	N/E
**Hearing and vestibular disorders**	0 (0%)	0 (0%)	0 (0%)	N/E	N/E
**Special senses other, disorders**	0 (0%)	1 (0.5%)	1 (0.5%)	N/E	N/E
**Psychiatric disorders**	3 (50%)	23 (11.7%)	26 (12.8%)	N/E	N/E
**Gastro-intestinal system disorders**	0 (0%)	79 (40.1%)	79 (38.9%)	N/E	N/E
**Liver and biliary system disorders**	2 (33.3%)	0 (0%)	2 (1.0%)	N/E	N/E
**Metabolic and nutritional disorders**	0 (0%)	5 (2.5%)	5 (2.5%)	N/E	N/E
**Endocrine disorders**	0 (0%)	0 (0%)	0 (0%)	N/E	N/E
**Cardiovascular disorders, general**	0 (0%)	0 (0%)	0 (0%)	N/E	N/E
**Heart rate and rhythm disorders**	0 (0%)	11 (5.6%)	11 (5.4%)	N/E	N/E
**Vascular (extracardiac) disorders**	0 (0%)	0 (0%)	0 (0%)	N/E	N/E
**Respiratory system disorders**	0 (0%)	2 (1.0%)	2 (1.0%)	N/E	N/E
**Red blood cell disorders**	0 (0%)	0 (0%)	0 (0%)	N/E	N/E
**White cell and RES* disorders**	0 (0%)	0 (0%)	0 (0%)	N/E	N/E
**Platelet, bleeding & clotting disorders**	0 (0%)	0 (0%)	0 (0%)	N/E	N/E
**Urinary system disorders**	0 (0%)	1 (0.5%)	1 (0.5%)	N/E	N/E
**Reproductive disorders, female**	0 (0%)	0 (0%)	0 (0%)	N/E	N/E
**Foetal disorders**	0 (0%)	0 (0%)	0 (0%)	N/E	N/E
**Neoplasms**	0 (0%)	0 (0%)	0 (0%)	N/E	N/E
**Body as a whole - general disorders**	1 (16.7%)	8 (4.1%)	9 (4.4%)	N/E	N/E
**Application site disorders**	0 (0%)	4 (2.0%)	4 (2.0%)	N/E	N/E
**Secondary terms - events**	0 (0%)	0 (0%)	0 (0%)	N/E	N/E
**Poison specific terms**	0 (0%)	0 (0%)	0 (0%)	N/E	N/E

Table S8. Association between System Organ Class-based adverse events and gender reported by the nurses

	AEs in Male (n=33)	AEs in Female (n=170)	Total AEs (n=203)	*P*-values	ROR (95% CI)
**Skin and appendages disorders**	1 (3.0%)	20 (11.8%)	21 (10.3%)	N/E	N/E
**Musculo-skeletal system disorders**	0 (0%)	5 (2.9%)	5 (2.5%)	N/E	N/E
**Central & peripheral nervous system disorders**	3 (9.1%)	32 (18.8%)	35 (17.2%)	N/E	N/E
**Vision disorders**	1 (3.0%)	1 (0.6%)	2 (1.0%)	N/E	N/E
**Hearing and vestibular disorders**	0 (0%)	0 (0%)	0 (0%)	N/E	N/E
**Special senses other, disorders**	0 (0%)	1 (0.6%)	1 (0.5%)	N/E	N/E
**Psychiatric disorders**	7 (21.2%)	19 (11.2%)	26 (12.8%)	>0.05	2.140 (0.818 – 5.596)
**Gastro-intestinal system disorders**	14 (42.4%)	65 (38.2%)	79 (38.9%)	>0.05	1.190 (0.559-2.536)
**Liver and biliary system disorders**	0 (0%)	1 (0.6%)	1 (0.5%)	N/E	N/E
**Metabolic and nutritional disorders**	2 (6.1%)	3 (1.8%)	5 (2.5%)	N/E	N/E
**Endocrine disorders**	0 (0%)	0 (0%)	0 (0%)	N/E	N/E
**Cardiovascular disorders, general**	0 (0%)	0 (0%)	0 (0%)	N/E	N/E
**Heart rate and rhythm disorders**	2 (6.1%)	9 (5.3%)	11 (5.4%)	N/E	N/E
**Vascular (extracardiac) disorders**	0 (0%)	0 (0%)	0 (0%)	N/E	N/E
**Respiratory system disorders**	0 (0%)	2 (1.2%)	2 (1.0%)	N/E	N/E
**Red blood cell disorders**	0 (0%)	0 (0%)	0 (0%)	N/E	N/E
**White cell and RES* disorders**	0 (0%)	0 (0%)	0 (0%)	N/E	N/E
**Platelet, bleeding & clotting disorders**	0 (0%)	0 (0%)	0 (0%)	N/E	N/E
**Urinary system disorders**	0 (0%)	1 (0.6%)	1 (0.5%)	N/E	N/E
**Reproductive disorders, female**	0 (0%)	0 (0%)	0 (0%)	N/E	N/E
**Foetal disorders**	0 (0%)	0 (0%)	0 (0%)	N/E	N/E
**Neoplasms**	0 (0%)	0 (0%)	0 (0%)	N/E	N/E
**Body as a whole - general disorders**	3 (9.1%)	11 (6.5%)	14 (6.9%)	N/E	N/E
**Application site disorders**	0 (0%)	0 (0%)	0 (0%)	N/E	N/E
**Secondary terms - events**	0 (0%)	0 (0%)	0 (0%)	N/E	N/E
**Poison specific terms**	0 (0%)	0 (0%)	0 (0%)	N/E	N/E

Table S9. Association between system Organ Class-based adverse events and seriousness of ADE reported by the general public

	**Serious AEs (n=22)**	**Nonserious AEs (n=1109)**	**Total AEs (n=1131)**	***P*-values**	**ROR (95% CI)**
**Skin and appendages disorders**	0 (0%)	66 (6.0%)	66 (5.8%)	N/E	N/E
**Musculo-skeletal system disorders**	0 (0%)	7 (0.6%)	7 (0.6%)	N/E	N/E
**Central & peripheral nervous system disorders**	10 (45.5%)	115 (10.4%)	125 (11.1%)	**<0.05**	**7.203 (3.045-17.041)**
**Vision disorders**	2 (9.1%)	4 (0.4%)	6 (0.5%)	N/E	N/E
**Hearing and vestibular disorders**	0 (0%)	3 (0.3%)	3 (0.3%)	N/E	N/E
**Special senses other, disorders**	0 (0%)	10 (0.9%)	10 (0.9%)	N/E	N/E
**Psychiatric disorders**	0 (0%)	114 (10.3%)	114 (10.1%)	N/E	N/E
**Gastro-intestinal system disorders**	5 (22.7%)	307 (27.7%)	312 (28.6%)	>0.05	0.768 (0.281-2.101)
**Liver and biliary system disorders**	0 (0%)	0 (0%)	0 (0%)	N/E	N/E
**Metabolic and nutritional disorders**	0 (0%)	11 (1.0%)	11 (1.0%)	N/E	N/E
**Endocrine disorders**	0 (0%)	2 (0.2%)	2 (0.2%)	N/E	N/E
**Cardiovascular disorders, general**	0 (0%)	5 (0.5%)	5 (0.4%)	N/E	N/E
**Heart rate and rhythm disorders**	0 (0%)	28 (2.5%)	28 (2.5%)	N/E	N/E
**Vascular (extracardiac) disorders**	0 (0%)	3 (0.3%)	3 (0.3%)	N/E	N/E
**Respiratory system disorders**	0 (0%)	9 (0.8%)	9 (0.8%)	N/E	N/E
**Red blood cell disorders**	0 (0%)	1 (0.1%)	1 (0.1%)	N/E	N/E
**White cell and RES* disorders**	0 (0%)	3 (0.3%)	3 (0.3%)	N/E	N/E
**Platelet, bleeding & clotting disorders**	0 (0%)	0 (0%)	0 (0%)	N/E	N/E
**Urinary system disorders**	0 (0%)	11 (1.0%)	11 (1.0%)	N/E	N/E
**Reproductive disorders, female**	0 (0%)	15 (1.3%)	15 (1.4%)	N/E	N/E
**Foetal disorders**	0 (0%)	1 (0.1%)	1 (0.1%)	N/E	N/E
**Neoplasms**	0 (0%)	4 (0.4%)	4 (0.4%)	N/E	N/E
**Body as a whole - general disorders**	4 (18.2%)	72 (6.5%)	76 (6.7%)	**<0.05**	**3.207 (1.057-9.725)**
**Application site disorders**	0 (0%)	298 (26.9%)	298 (26.3%)	N/E	N/E
**Secondary terms - events**	1 (4.5%)	20 (1.8%)	21 (1.9%)	N/E	N/E
**Poison specific terms**	0 (0%)	0 (0%)	0 (0%)	N/E	N/E

Table S10. Association between System Organ Class-based adverse events and gender reported by the general public

	AEs in Male (n=95)	AEs in Female (n=930)	Total AEs (n=1025)	*P*-values	ROR (95% CI)
**Skin and appendages disorders**	5 (5.3%)	49 (5.3%)	54 (5.3%)	N/E	N/E
**Musculo-skeletal system disorders**	0 (0%)	7 (0.8%)	7 (0.7%)	N/E	N/E
**Central & peripheral nervous system disorders**	14 (14.7%)	99 (10.6%)	113 (11.0%)	>0.05	1.451 (0.793-2.655)
**Vision disorders**	1 (1.1%)	4 (0.4%)	5 (0.5%)	N/E	N/E
**Hearing and vestibular disorders**	0 (0%)	3 (0.3%)	3 (0.3%)	N/E	N/E
**Special senses other, disorders**	1 (1.1%)	4 (0.4%)	5 (0.5%)	N/E	N/E
**Psychiatric disorders**	9 (9.5%)	75 (8.1%)	84 (8.2%)	>0.05	1.193 (0.577-2.466)
**Gastro-intestinal system disorders**	36 (37.9%)	273 (29.4%)	309 (30.1%)	>0.05	1.468 (0.085-2.275)
**Liver and biliary system disorders**	1 (1.1%)	0 (0%)	1 (0.1%)	N/E	N/E
**Metabolic and nutritional disorders**	0 (0%)	9 (1.0%)	9 (0.9%)	N/E	N/E
**Endocrine disorders**	0 (0%)	2 (0.2%)	2 (0.2%)	N/E	N/E
**Cardiovascular disorders, general**	1 (1.1%)	3 (0.3%)	4 (0.4%)	N/E	N/E
**Heart rate and rhythm disorders**	2 (2.1%)	21 (2.3%)	23 (2.2%)	N/E	N/E
**Vascular (extracardiac) disorders**	0 (0%)	1 (0.1%)	1 (0.1%)	N/E	N/E
**Respiratory system disorders**	0 (0%)	10 (1.1%)	10 (1.0%)	N/E	N/E
**Red blood cell disorders**	0 (0%)	1 (0.1%)	1 (0.1%)	N/E	N/E
**White cell and RES* disorders**	0 (0%)	0 (0%)	0 (0%)	N/E	N/E
**Platelet, bleeding & clotting disorders**	0 (0%)	3 (0.3%)	3 (0.3%)	N/E	N/E
**Urinary system disorders**	2 (2.1%)	8 (0.9%)	10 (1.0%)	N/E	N/E
**Reproductive disorders, female**	0 (0%)	15 (1.6%)	15 (1.5%)	N/E	N/E
**Foetal disorders**	0 (0%)	1 (0.1%)	1 (1.0%)	N/E	N/E
**Neoplasms**	1 (1.1%)	1 (0.1%)	2 (0.2%)	N/E	N/E
**Body as a whole - general disorders**	11 (11.6%)	65 (7.0%)	76 (7.4%)	>0.05	1.746 (0.885-3.430)
**Application site disorders**	11 (11.6%)	256 (27.5%)	267 (26.0%)	**<0.05**	**0.345 (0.191-0.657)**
**Secondary terms - events**	0 (0%)	20 (2.2%)	20 (2.0%)	N/E	N/E
**Poison specific terms**	0 (0%)	0 (0%)	0 (0%)	N/E	N/E

Table S11. Association between system Organ Class-based adverse events and seriousness of ADE reported by the others

	Serious AEs (n=13)	Nonserious AEs (n=111)	Total AEs (n=124)	*P*-values	ROR (95% CI)
**Skin and appendages disorders**	0 (0%)	19 (17.1%)	19 (15.3%)	N/E	N/E
**Musculo-skeletal system disorders**	0 (0%)	1 (0.9%)	1 (0.8%)	N/E	N/E
**Central & peripheral nervous system disorders**	0 (0%)	15 (13.5%)	15 (12.1%)	N/E	N/E
**Vision disorders**	0 (0%)	0 (0%)	0 (0%)	N/E	N/E
**Hearing and vestibular disorders**	0 (0%)	0 (0%)	0 (0%)	N/E	N/E
**Special senses other, disorders**	0 (0%)	0 (0%)	0 (0%)	N/E	N/E
**Psychiatric disorders**	9 (69.2%)	5 (4.5%)	14 (11.3%)	**<0.05**	**47.7 (10.853-209.637)**
**Gastro-intestinal system disorders**	1 (7.7%)	22 (19.8%)	23 (18.5%_	N/E	N/E
**Liver and biliary system disorders**	0 (0%)	0 (0%)	0 (0%)	N/E	N/E
**Metabolic and nutritional disorders**	0 (0%)	2 (1.8%)	2 (1.6%)	N/E	N/E
**Endocrine disorders**	0 (0%)	0 (0%)	0 (0%)	N/E	N/E
**Cardiovascular disorders, general**	0 (0%)	0 (0%)	0 (0%)	N/E	N/E
**Heart rate and rhythm disorders**	0 (0%)	0 (0%)	0 (0%)	N/E	N/E
**Vascular (extracardiac) disorders**	1 (7.7%)	0 (0%)	1 (0.8%)	N/E	N/E
**Respiratory system disorders**	0 (0%)	2 (1.8%)	2 (1.6%)	N/E	N/E
**Red blood cell disorders**	0 (0%)	0 (0%)	0 (0%)	N/E	N/E
**White cell and RES* disorders**	0 (0%)	0 (0%)	0 (0%)	N/E	N/E
**Platelet, bleeding & clotting disorders**	0 (0%)	0 (0%)	0 (0%)	N/E	N/E
**Urinary system disorders**	0 (0%)	0 (0%)	0 (0%)	N/E	N/E
**Reproductive disorders, female**	0 (0%)	2 (1.8%)	2 (1.6%)	N/E	N/E
**Foetal disorders**	0 (0%)	0 (0%)	0 (0%)	N/E	N/E
**Neoplasms**	0 (0%)	0 (0%)	0 (0%)	N/E	N/E
**Body as a whole - general disorders**	0 (0%)	9 (8.1%)	9 (7.3%)	N/E	N/E
**Application site disorders**	0 (0%)	33 (29.7%)	33 (26.6%)	N/E	N/E
**Secondary terms - events**	2 (15.4%)	1 (0.9%)	3 (2.4%)	N/E	N/E
**Poison specific terms**	0 (0%)	0 (0%)	0 (0%)	N/E	N/E

Table S12. Association between System Organ Class-based adverse events and gender reported by the others

	AEs in Male (n=13)	AEs in Female (n=48)	Total AEs (n=61)	*P*-values	ROR (95% CI)
**Skin and appendages disorders**	2 (15.4%)	5 (10.4%)	7 (11.5%)	N/E	N/E
**Musculo-skeletal system disorders**	0 (0%)	1 (2.1%)	1 (1.64%)	N/E	N/E
**Central & peripheral nervous system disorders**	0 (0%)	11 (22.9%)	11 (18.0%)	N/E	N/E
**Vision disorders**	0 (0%)	0 (0%)	0 (0%)	N/E	N/E
**Hearing and vestibular disorders**	0 (0%)	0 (0%)	0 (0%)	N/E	N/E
**Special senses other, disorders**	0 (0%)	0 (0%)	0 (0%)	N/E	N/E
**Psychiatric disorders**	6 (46.2%)	8 (16.7%)	14 (23.0%)	**<0.05**	**4.286 (1.135-16.182)**
**Gastro-intestinal system disorders**	1 (7.7%)	15 (31.3%)	16 (26.2%)	N/E	N/E
**Liver and biliary system disorders**	0 (0%)	0 (0%)	0 (0%)	N/E	N/E
**Metabolic and nutritional disorders**	0 (0%)	1 (2.1%)	1(1.6%)	N/E	N/E
**Endocrine disorders**	0 (0%)	0 (0%)	0 (0%)	N/E	N/E
**Cardiovascular disorders, general**	0 (0%)	0 (0%)	0 (0%)	N/E	N/E
**Heart rate and rhythm disorders**	0 (0%)	0 (0%)	0 (0%)	N/E	N/E
**Vascular (extracardiac) disorders**	0 (0%)	1 (2.1%)	1 (1.6%)	N/E	N/E
**Respiratory system disorders**	0 (0%)	2 (4.2%)	2 (3.3%)	N/E	N/E
**Red blood cell disorders**	0 (0%)	0 (0%)	0 (0%)	N/E	N/E
**White cell and RES* disorders**	0 (0%)	0 (0%)	0 (0%)	N/E	N/E
**Platelet, bleeding & clotting disorders**	0 (0%)	0 (0%)	0 (0%)	N/E	N/E
**Urinary system disorders**	0 (0%)	0 (0%)	0 (0%)	N/E	N/E
**Reproductive disorders, female**	0 (0%)	1 (2.1%_	1 (1.64%)	N/E	N/E
**Foetal disorders**	0 (0%)	0 (0%)	0 (0%)	N/E	N/E
**Neoplasms**	0 (0%)	0 (0%)	0 (0%)	N/E	N/E
**Body as a whole - general disorders**	2 (15.4%)	3 (6.25%)	5 (8.2%)	N/E	N/E
**Application site disorders**	0 (0%)	0 (0%)	0 (0%)	N/E	N/E
**Secondary terms - events**	2 (15.4%)	0 (0%)	2 (3.3%)	N/E	N/E
**Poison specific terms**	0 (0%)	0 (0%)	0 (0%)	N/E	N/E
